# Melatonin Accumulation in Sweet Cherry and Its Influence on Fruit Quality and Antioxidant Properties

**DOI:** 10.3390/molecules25030753

**Published:** 2020-02-10

**Authors:** Hui Xia, Yanqiu Shen, Tian Shen, Xin Wang, Xuefeng Zhang, Peng Hu, Dong Liang, Lijin Lin, Honghong Deng, Jin Wang, Qunxian Deng, Xiulan Lv

**Affiliations:** 1 College of Horticulture, Sichuan Agricultural University, Chengdu 611130, China; susanxia_2001@163.com (H.X.); s2020sprilynn@163.com (Y.S.); st13992494739@163.com (T.S.); wx13458193919@163.com (X.W.); zxf012138@163.com (X.Z.); hupengniubi@sohu.com (P.H.); llj800924@163.com (L.L.); denghonghong2010@163.com (H.D.); wjin@sicau.edu.cn (J.W.); dqxlwj@sina.com (Q.D.); xllvjj@163.com (X.L.); 2 Institute of Pomology and Olericulture, Sichuan Agricultural University, Chengdu 611130, China

**Keywords:** sweet cherry, melatonin, biosynthesis, exogenous application, fruit quality

## Abstract

Although the effects of melatonin on plant abiotic and biotic stress resistance have been explored in recent decades, the accumulation of endogenous melatonin in plants and its influence on fruit quality remains unclear. In the present study, melatonin accumulation levels and the expression profiles of five synthesis genes were investigated during fruit and leaf development in sweet cherry (*Prunus avium* L.). Melatonin was strongly accumulated in young fruits and leaves, then decreased steadily with maturation. Transcript levels of *PacTDC* and *PacSNAT* were highly correlated with melatonin content in both fruit and leaves, indicating their importance in melatonin accumulation. Furthermore, application of 50 and 100 μmol·L^−1^ of melatonin to leaves had a greater influence on fruit quality than treatments applied to fruits, by significantly improving fruit weight, soluble solids content, and phenolic content including total phenols, flavanols, total anthocyanins, and ascorbic acid. Meanwhile, melatonin application promoted the antioxidant capacity of fruit assayed by 2,2-diphenyl-1-picrylhydrazyl (DPPH), 2,2′-azinobis (3-ethylben zothiazoline-6-sulfonic acid) (ABTS), and ferric reducing antioxidant power (FRAP). These results provide insights into the physiological and molecular mechanisms underlying melatonin metabolism of sweet cherry.

## 1. Introduction

Melatonin (*N*-acetyl-5-methoxytryptamine), originally identified from the bovine pineal gland, has beneficial effects on human health, such as regulating circadian rhythms, metabolism, and the immune system [1]. Since melatonin was first discovered in plants in 1995 [2,3], its functional role in plants has been extensively explored [4]. Substantial evidence suggests that melatonin, as a free radical scavenger in vitro or in vivo, provides resilience to various biotic and abiotic stresses, including drought, salinity, chilling, heat, heavy metals, ultraviolet radiation, pathogens, iron deficiency, and herbicides [5,6,7,8,9,10,11,12,13,14,15]. Moreover, as a growth regulator, melatonin is involved in the regulation of plant development, including ion uptake [16], opening and/or closing of stomata [17], root formation [18], seed germination [19], photosynthesis [20], senescence [21,22], and photoperiod response [23]. 

Recently, exogenous melatonin treatment has been tested for its ability to promote ripening and improve fruit quality postharvest, and has been shown to improve postharvest quality of strawberry [24]; ameliorate chilling tolerance in pomegranate [25], peach [26], and tomato [27]; and attenuate postharvest decay and maintain the nutritional quality of strawberry fruit [28]. However, little information is available regarding endogenous melatonin accumulation in plants and the effect of melatonin on fruit development. 

In plants, the melatonin biosynthesis pathway has recently been revealed, and chloroplasts and mitochondria have been proposed as the major sites of biosynthesis [29]. Melatonin is synthesized from tryptophan by four series enzymes: tryptophan decarboxylase (TDC); tryptamine 5-hydroxylase (T5H); serotonin *N*-acetyltransferase (SNAT); and *N*-acetylserotonin O-methyltransferase (ASMT) [30,31]. Sequences and expression patterns of genes involved in melatonin biosynthesis have been identified in several plants including rice [32], *Arabidopsis thaliana* [33], loblolly pine [34], cherry [35], apple [36], and cassava [37]. However, there are few systematic studies of melatonin synthesis genes in plants.

Sweet cherry (*Prunus avium* L.) is a popular and economically valuable fruit cultivated in temperate regions of the world and is recognized for its nutraceutical properties and antioxidant activity. Cherries have a relatively higher melatonin content than other fruits [38,39,40], which makes them ideal for studying melatonin accumulation in plants. The aim of the present study was to investigate the endogenous melatonin accumulation in sweet cherry and the expression patterns of five melatonin synthesis genes during fruit development. In addition, the effects of exogenous melatonin application on fruit qualities and antioxidant capacity were evaluated using three different melatonin concentrations sprayed on the fruit or leaves. Our results provide insights into the physiological and molecular mechanisms underlying melatonin metabolism of sweet cherry.

## 2. Results

### 2.1. Melatonin Accumulation in Fruits and Leaves During Development 

**Figure 1:**
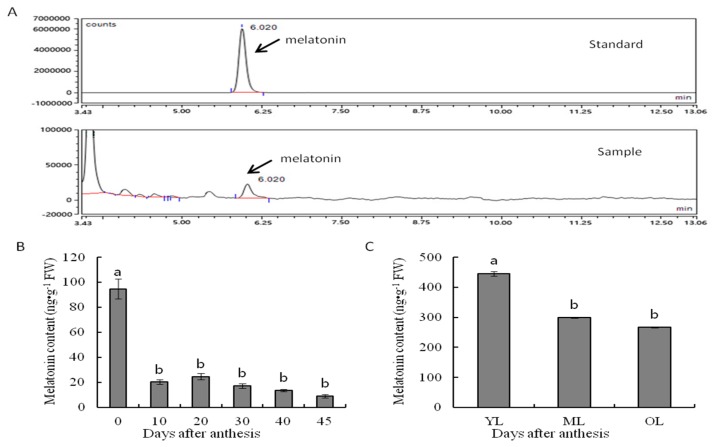
(**A**) HPLC chromatogram for melatonin and its concentrations in (**B**) fruit and (**C**) leaves of Hongdeng. YL: young leaves; ML: mature leaves; OL: old leaves. Data are shown as mean ± SE with five biological replicates, different letters indicate significant differences at *p* < 0.05 level.

The endogenous melatonin concentrations in fruit and leaves were detected by HPLC equipped with a fluorescence detector (FLD) and melatonin peaked at 6.02 min; standard and sample results are shown in Figure 1A. The highest concentration of melatonin was detected in young fruit just after flower fall (0 d). The concentration then decreased dramatically to approximately one-fourth of the initial value at 10 d, before increasing slightly at 20 d then decreasing gradually until maturity (Figure 1B). Similarly, young leaves displayed the highest level of melatonin concentration, followed by mature and senescent leaves (Figure 1C). 

### 2.2. Expression Profiles of Melatonin Synthesis Genes in Fruit

**Figure 2:**
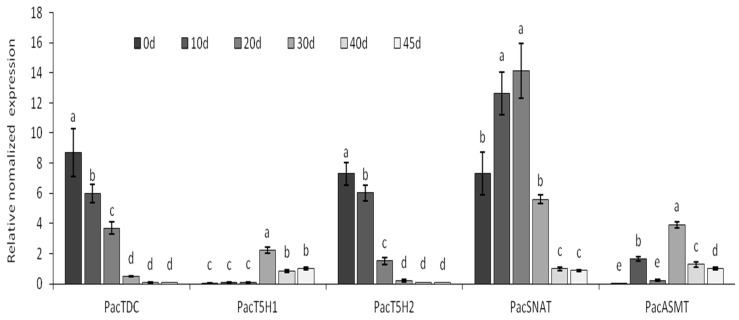
The transcriptional expression level of *PacTDC, PacT5H1, PacT5H2, PacSNAT,* and *PacASMT* during fruit development in Hongdeng. Data are shown as mean ± SE with three biological replicates, different letters indicate significant differences at *p* < 0.05 level.

In this study, qRT-PCR was used to investigate the expression profiles of five melatonin synthesis genes during fruit development, namely *PacTDC, PacT5H1, PacT5H2, PacSNAT,* and *PacASMT*, with *actin* and *EF2* serving as the internal standards (Figure 2). 

The expression levels of *PacTDC* and *PacT5H2* both decreased rapidly for 30 d then decreased slowly until maturity which was consistent with the pattern of melatonin concentration change. The expression of *PacSNAT* increased at first, peaked at 30 d, then decreased rapidly. *PavT5H1* exhibited a relatively low expression level compared with that of the other genes. The expression of *PacASMT* peaked at 30 d, then decreased. 

### 2.3. Expression Level of Melatonin Synthesis Genes in Leaves

**Figure 3:**
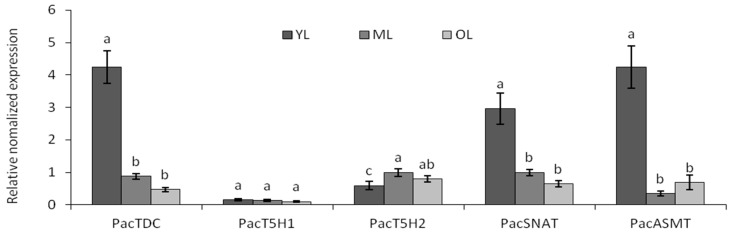
The transcriptional expression level of *PacTDC, PacT5H1, PacT5H2, PacSNAT*, and *PacASMT* during leaf development in Hongdeng. YL: young leaves; ML: mature leaves; OL: old leaves. Data are shown as mean ± SE with three biological replicates, different letters indicate significant differences at *p* < 0.05 level.

*PacTDC* and *PacSNAT* had the highest expression levels at 0 d, which then decreased significantly with leaf development (Figure 3); this pattern was consistent with the change in melatonin content. The relative mRNA expression of *PacASMT* was the highest in young leaves and lower in mature and old leaves. *PacT5H1* exhibited the lowest expression level in leaves compared with that of other genes, which was similar to the results for fruit. Additionally, the mature leaves had higher *PacT5H2* transcriptional level than young or senescent leaves. 

### 2.4. Melatonin Application Improves Fruit Quality

To better understand the function of melatonin in fruit ripening and quality improvement, a series of fruit quality features were investigated in fruit from the control 0 μmol·L^−1^ (CK) and different melatonin treatment groups 50 μmol·L^−1^ (50MT), 100 μmol·L^−1^ (100MT), or 200 μmol·L^−1^ (200MT) application on fruits and leaves.

**Figure 4:**
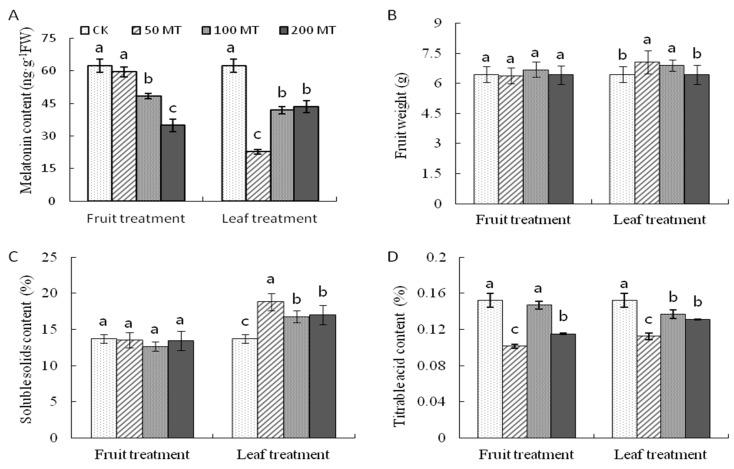
(**A**) The melatonin content in fruits, and the changes of (**B**) fruit weight, (**C**) soluble solids content, and (**D**) titrable acid content after exogenous melatonin application in Hongdeng sweet cherry. Data are shown as mean ± SE with five biological replicates, different letters indicate significant differences at *p* < 0.05 level.

Melatonin application decreased the endogenous melatonin concentration regardless of whether the fruit or leaves were sprayed (Figure 4A). The endogenous melatonin concentration decreased as the treatment concentration increased with fruit spraying, but it increased with treatment concentration with leaf spraying.

In the fruit spraying treatments, there was no significant difference in the fruit weight (Figure 4B) or soluble solids content (SSC) (Figure 4C) between the CK and MT treatments. In the leaf spraying treatment, fruit weight and SSC of fruits from the 50MT and 100MT treatments were higher than those for the control (Figure 4B,C). Regardless of treatment type (fruit or leaf), exogenous melatonin was responsible for reducing the titrable acid (TA) content of fruits, especially in the 50MT and 200MT treatments (Figure 4D).

### 2.5. Exogenous Melatonin Improves Antioxidant Content of Fruit

**Figure 5:**
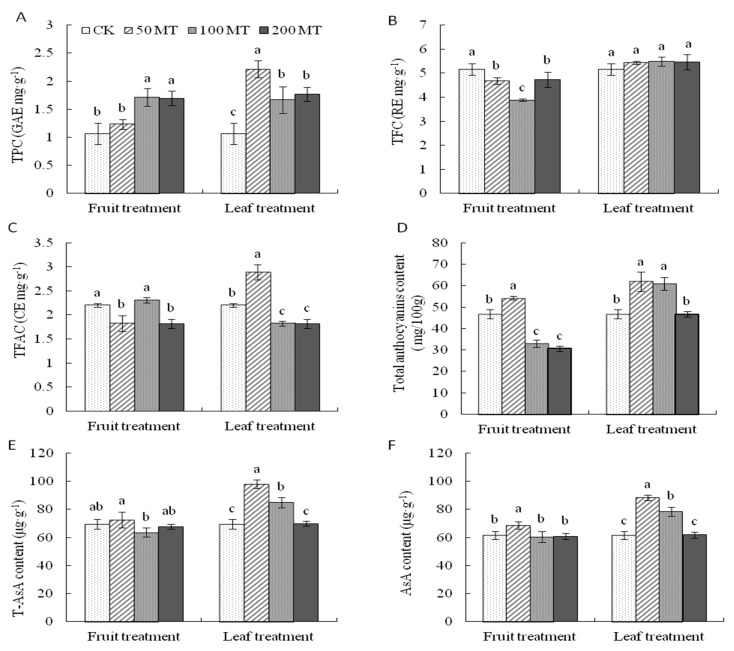
The changes of (**A**) total phenolics content (TPC), (**B**) total flavonoids content (TFC), (**C**) total flavanols content (TFAC), (**D**) total monomeric anthocyanins, (**E**) total ascorbic acid, and (**F**) ascorbic acid after exogenous melatonin application in Hongdeng sweet cherry. Data are shown as mean ± SE, with five biological replicates, different letters indicate significant differences at *p* < 0.05 level.

Exogenous melatonin application influenced secondary metabolites in sweet cherry fruits. Total phenolics content (TPC) was improved with all melatonin treatments when compared with that of the control; leaf spraying had a greater effect than fruit spraying, especially when spraying with 50MT (2.5 fold increase) (Figure 5A).

Spraying melatonin on the fruit significantly decreased the total flavonoids content (TFC), and 100MT resulted in the lowest value, while leaf spraying improved TFC in fruit but not significantly compared with that of the control (Figure 5B). Exogenous melatonin application improved the total flavanols content (TFAC) of berries. In the fruit spraying treatments, fruits from 100MT contained the highest TFAC (2.31 mg CE/g FW) while the lowest values were found in 50MT and 200MT. However, for the leaf spraying treatments, the TFAC of fruits from 50MT was significantly higher than that of the control, 100MT, and 200MT (Figure 5C).

Melatonin application on fruit did not change the total anthocyanin content, except at 50MT. However, leaf spraying with 50MT and 100MT significantly increased the total anthocyanin content compared with that of CK (Figure 5D). In addition, the 50MT treatment (fruit and leaf spraying) increased the content of total ascorbic acid (T-AsA) (Figure 5E) and AsA (11.6% and 43.6% in the fruit and leaf treatments, respectively) (Figure 5F).

### 2.6. Exogenous Melatonin Improves Antioxidant Activities of Sweet Cherry Fruit

**Figure 6:**
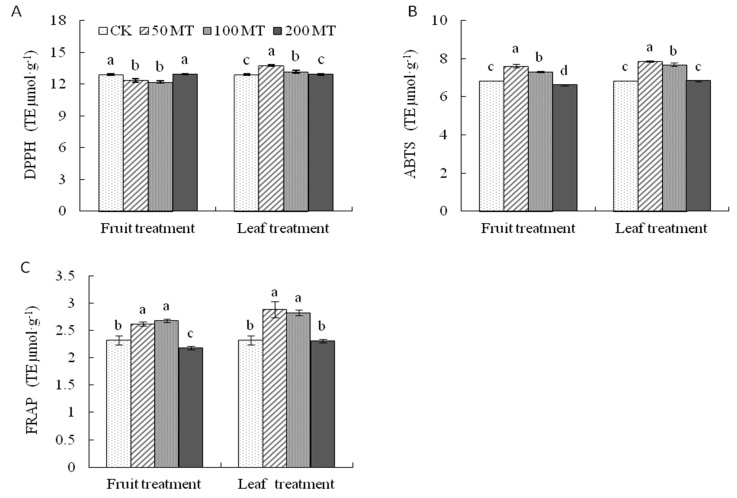
Antioxidant activity determined by (**A**) 2,2-diphenyl-1-picrylhydrazyl (DPPH), (**B**) 2,2′-azinobis (3-ethylben zothiazoline-6-sulfonic acid) (ABTS), and (**C**)ferric reducing antioxidant power (FRAP) assays of Hongdeng sweet cherry after exogenous melatonin application. Data are shown as mean ± SE, with five biological replicates, different letters indicate significant differences at *p* < 0.05 level.

Three antioxidant assays 2,2-diphenyl-1-picrylhydrazyl (DPPH), 2,2′-azinobis (3-ethylben zothiazoline-6-sulfonic acid) (ABTS), and ferric reducing antioxidant power (FRAP) were applied to reflect the antioxidant activities in the fruit and leaf treatments in this study. In the fruit treatment, the DPPH radical-scavenging capacity decreased in the order CK, 200MT < 50MT, 100MT(Figure 6A). However, in the leaf treatment, the same activity decreased in the order 50MT < 100MT < 200MT, CK. The highest ABTS (Figure 6B) inhibition value was measured in the 50MT sample, followed by 100MT, CK, and 200MT in the fruit treatment. In addition, in the leaf treatment, the maximum ABTS inhibition was measured in the 50MT sample, which was significantly higher than that in the other treatments. For both fruit and leaf spraying, exogenous melatonin application (50MT and 100MT) sharply increased the FRAP (Figure 6C) of fruits by 12.88% and 22.75% (fruit treatment) and 24.03% and 21.46% (leaf treatment), respectively, compared with that of the control, while 200MT showed the inverse pattern.

## 3. Discussion

In recent years, studies on melatonin have focused on its exogenous application to improve plant tolerance to various abiotic and biotic stresses or maintain fruit quality postharvest. However, little is known about the putative role of melatonin in sweet cherry fruit development (but see Zhao et al. [35] and Tijero et al. [41]). Our results showed that endogenous melatonin decreased during fruit ripening in sweet cherry. This result is in accordance with those of Tijero et al. [41], who concluded that melatonin played an inhibitory role during sweet cherry ripening and has a delicate hormonal balance with abscisic acid, salicylic acid, and jasmonic acid [41]. Zhao et al. [35] found that the melatonin content in stage II of fruit development was higher than that in stages I and Ш. Together, these results indicate that the melatonin concentration in plant cells is highly regulated by developmental processes.

Kang et al. [42] reported that four critical enzymes (TDC, T5H, SNAT, and ASMT) synthesize melatonin in rice. Melatonin biosynthetic and metabolic pathways have been revealed in many plants. In the present study, we identified five melatonin synthesis genes in sweet cherry: *PacTDC, PacT5H1, PacT5H2, PacSNAT*, and *PacASMT.* Expression levels of *PacTDC*, *PacSNAT,* and *PacASMT* decreased during fruit development with the ripening process, which is consistent with observations of *PacTDC* in sweet cherry [35] and *MdSNAT* in apple [36]. Byeon et al. [32] demonstrated that melatonin synthesis was counter-regulated by *SNAT* in rice. In loblolly pine, the *SNAT* mRNA levels were not similar to those of melatonin content in different tissues, and SNAT protein was only abundant in leaves [34], suggesting that SNAT is the key biosynthetic enzyme in melatonin biosynthesis and degradation. Thus, a putative reason for the discrepancy was the translational regulation of *SNAT*. ASMT is the last enzyme in melatonin biosynthesis. Over-expression of *ASMT* in *Malus zumi* improved melatonin production and enhanced drought tolerance in transgenic *Arabidopsis thaliana* [43]. The Cd-induced synthesis of melatonin coincided with increased expression of *ASMT* in rice [44]. Transient expression of cassava *ASMT* in tobacco leaves increased the melatonin content [45]. *ASMT* in pepper fruit tissues (placenta, seeds, and pericarp) had higher transcriptional levels in the early stages of fruit development [46]. In our results, melatonin synthesis during fruit development coincided with increased expression of *PacASMT*, suggesting that this gene may contribute to melatonin accumulation in fruits.

There are few studies of melatonin biosynthesis and related gene expression in leaf development. The melatonin content of buds was significantly lower than that of leaves of loblolly pine [34]. Endogenous melatonin concentrations increased gradually in parallel with development toward leaf senescence in *Arabidopsis* [47]. In contrast, the melatonin content decreased during leaf development in this study. The reason for this discrepancy is not clear, but may be due to different species and growth conditions. In addition, transcriptional patterns of *PacTDC, PacSNAT, and PacASMT* were consistent with changes in melatonin content, which indicates that they play key roles in melatonin biosynthesis in leaves, as seen in fruit in this study.

Previous studies have highlighted the effect of exogenous melatonin on fruit qualities. Meng et al. [48] showed that melatonin treatment of pre-veraison grape berries increased the weight of the berries at maturity and harvest. Melatonin may also promote postharvest fruit ripening and anthocyanin accumulation in tomato [49]. Exogenous melatonin plus gibberellic acid application increased the number, weight, length, SSC, TP, and TFC of jumbo blackberry fruit [50]. However, high levels of melatonin inhibited the growth of apple and induced significant accumulation of fructose, glucose, and sucrose in the leaves [51]. In this study, 50MT or/and 100MT concentrations improved the weight, SSC, TA, and secondary metabolites in sweet cherry fruits. Based on the above results, it seems that melatonin has a positive effect on different fruits during maturation by mediating metabolism, including glycometabolism and flavonoid biosynthesis. However, Tijero et al. [41] found that exogenous melatonin treatment delayed anthocyanin accumulation, thus confirming an inhibitory regulatory role for melatonin in fruit ripening. Therefore, we infer that melatonin may modulate fruit ripening in different fruits depending on its concentration, application time, etc. Additional research is needed to understand the functions of melatonin in determining fruit quality.

Recently, studies have suggested that melatonin acts as an antioxidant, a biostimulator, and a plant growth regulator during plant development and responses to abiotic stress [52]. There are two ways these results show how melatonin is possibly a powerful antioxidant. First, melatonin may directly enhance antioxidant ability as an antioxidant molecule. Second, melatonin application enhanced antioxidant content, for example, flavonoids and AsA, suggesting that melatonin may work as a biostimulator to regulate biosynthesis of antioxidants [31]. In this study, melatonin application enhanced the antioxidant content of sweet cherry fruits, including TPC, TFC, TFAC, TMAC, and AsA (Figure 5), which has also been found in grape [53], tomato [54], and date palm [55]. Therefore, melatonin application enhanced plant antioxidant ability in the present study.

## 4. Materials and Methods

### 4.1. Plant Material

Fruit samples were collected from an eight-year-old sweet cherry (*Prunus avium* L.) cultivar Hongdeng in the orchard of Sichuan Agricultural University, Ya’an, Sichuan, China (29.5° N, 102.62° E) in 2017 and 2018 with an average maximum temperature range from 22–25 °C and average minimum temperature range from 7–15 °C during the fruit development period (April to June), the details are shown in Table S1. After anthesis, 75%–80% flower fall was set as day 0 and at least fifty fruits were picked at 0, 10, 20, 30, 40, and 45 d. When fruits were harvested, the leaves at the top, middle, and base of 10 similar vegetative shoots from Hongdeng were collected as the young, mature, and old leaves.

Just before the fruits turned red, the leaves (leaf treatment) or fruits (fruit treatment) were sprayed with different concentrations of melatonin solution: 0 μmol·L^−1^ (CK), 50 μmol·L^−1^ (50MT), 100 μmol·L^−1^ (100MT), or 200 μmol·L^−1^ (200MT), three times at seven-day intervals. When the fruits matured, 50 berries from every treatment were harvested to assess the nutraceutical properties and fruit quality.

### 4.2. Determination of Melatonin Concentration

To determine melatonin accumulation, we used the modified methods of González-Gómez [56]. In brief, 0.5 g samples were weighed and ground into a homogenate in 5 mL methanol, which was then extracted using 200 W ultrasonic oscillation for 30 min and centrifuged at 10,000 r·min^−1^ for 15 min. Two milliliters of supernatant was taken and filtered with a 0.22 μm organic filter membrane for melatonin assay using high-performance liquid chromatography (HPLC).

An HPLC system (Agilent 1260; Agilent Technologies, Santa Clara, CA, USA) equipped with an FLD detector and an Inertsil ODS-3 C18 column (5 μm, 250 mm × 4.6 mm; GL Sciences B.V., Eindhoven, The Netherlands) was used to determine the melatonin concentration, with a mobile phase of water, methanol, and acetic acid (ratio 44.9:55:0.1) at a flow rate of 0.8 mL·min^−1^. Melatonin was detected at 280 nm excitation and 384 nm emission wavelength.

### 4.3. RNA Extraction and qRT-PCR

Total RNA was extracted from samples by the modified cetyltrimethylammonium bromide (CTAB)method [57]. The degenerate primers of *PacTDC*, *PacT5H1*, *PacT5H2*, *PacSNAT*, and *PacASMT* were designed (Table S1) based on our transcriptome data (unpublished) after blastP analysis with homologous genes of other species and the sweet cherry genome (Shirasawa et al., 2017). The candidate sequences with the highest identity were cloned from Hongdeng cDNA using reverse transcription PCR.

Quantitative PCR (qPCR) was used to analyze the transcript levels of genes involved in the synthesis of melatonin in the different treatments. Primer3 (http://bioinfo.ut.ee/primer3-0.4.0/primer3/) was used to design the primers (Table S2). One microgram total RNA was used to synthesize the first strand of cDNA using the PrimeScriptTM RT Reagent Kit with gDNA Eraser (Perfect Real Time) (TaKaRa Bio Inc., Kusatsu, Japan). The synthesis was performed according to the manufacturer’s instructions. qPCR was performed with a SYBR Premix Ex Taq^TM^ II Kit (TaKaRa) on a Real-Time System (CFX96; Bio-Rad Laboratories, Irvine, CA, USA). As an internal control, elongation factor 2 (*EF2*) and *actin* (Table S1) were used to normalize the relative expression levels of the genes studied. Three PCR replicates were conducted per sample and the 2^-ΔΔCt^ method was applied to calculate the relative expression levels.

### 4.4. Fruit Quality Evaluation

Freshly picked bunches of berries were weighed using an analytical balance with a precision of 0.1 g. Juice characteristics were determined from a sample of 50 berries per plot: the soluble solids content (SSC) was measured using a pocket-refractometer (PAL-1; Atago Instruments, Tokyo, Japan). Titratable acidity (TA) was determined by titration with 0.1 N NaOH using 5 mL of diluted juice and expressed as g tartaric acid 100 mL^−1^ juice.

### 4.5. Phenolic Compounds, Total Anthocyanin, and AsA Content

The content of total phenolics (TPC), flavonoids (TFC), flavanols (TFAC), and total anthocyanins were measured using the methods described by Liang et al. [22]. In brief, 0.2 g of leaf tissue was ground as a homogenate with cold 70% (*v/v*) methanol containing 2% (*v/v*) formic acid and 28% (*v/v*) ethanol. The homogenate was ultrasonically extracted for 30 min and shaken at 250 rpm for 2 h at 30 °C. Then the homogenate was centrifuged at 10,000× *g* for 10 min at 4 °C and the supernatant was filtered using a 0.45 mm filter membrane for further analysis. TPC was determined using the Folin–Ciocalteu method and the absorbance was measured using an ultraviolet spectrophotometer at 765 nm with gallic acid as the standard; the result was expressed as gallic acid equivalent. The absorption of TFC was measured at 510 nm and expressed as rutin equivalent. TFAC was determined using the HCl-acidified 4-dimethylaminocinnamaldehyde (p-DMACA) method; absorbance was determined at 640 nm and the result was expressed as catechin equivalent.

The pH differential was used to measure total anthocyanin content. The fruit extract was diluted to pH 1.0 and 4.5 using a buffer solution and the absorbance values at 510 and 700 nm were measured at each pH; the total anthocyanin content was calculated as the difference between them. Ascorbic acid (AsA) and T-AsA content were measured using the methods of Li [58].

### 4.6. Antioxidant Capacity

The free radical scavenging ability, including 2,2-diphenyl-1-picrylhydrazyl (DPPH), 2,2′-azinobis (3-ethylben zothiazoline-6-sulfonic acid) (ABTS), and ferric reducing antioxidant power (FRAP) were measured using the methods described by Xu and Chen [59]. All results were expressed as micromole trolox equivalent per gram of freeze-dried sample (μmol TE/g FDW). The above indices were measured more than three times, and the average value was taken as the measured value of each treatment.

### 4.7. Statistical Analysis

Excel 2010 was used for data processing. Analysis of variance was performed using SPSS 22.0. Significant differences were detected by Duncan’s multiple range tests at the *p* < 0.05 level.

## 5. Conclusions

Endogenous melatonin accumulated in young fruit and leaf tissues then decreased steadily toward maturation. The expression of melatonin biosynthesis genes indicated that *PacSNAT* might play a key regulatory role in melatonin biosynthesis at the transcriptional level. In addition, exogenous melatonin application improved the nutraceutical properties of sweet cherry fruits, especially when sprayed on leaves at a concentration of 50 μmol·L^−1^. These results can help in revising agricultural procedures to reinforce the content of antioxidant melatonin in mature fruit used in human diets.

## Supplementary Materials

The following are available online, Table S1: Temperature changes in Ya’an City during fruit development in 2017 and 2018, Table S2: Primers used in this study.
