# Supplementary material for: Melatonin Accumulation in Sweet Cherry and Its Influence on Fruit Quality and Antioxidant Properties
Source: Molecules. 2020 Feb 10;25(3):753. doi: 10.3390/molecules25030753 (PMC7037775; doi:10.3390/molecules25030753)
Supplement: Supplementary file 1 [file molecules-25-00753-s001.pdf]

molecules
MDPI

Supplementary Materials

Table S1 Temperature changes in Ya'an City during fruit development in 2017 and 2018 (°C)

	2017			2018		
	April	May	June	April	May	June
Average maximum temperature	23	25	24	22	24	25
Average minimum temperature	13	16	17	13	16	18
Maximum temperature	31	32	30	28	32	31
Minimum temperature	10	12	13	7	13	15

Table S2 Primers used in this study

Name	Sequence	Usage
TDCs	ctacttcttggtttcctttc	full-length cDNA cloning
TDCa	tcaacatattattgtgcttt	
T5Hs	ggtggctaccttgcctatga	full-length cDNA cloning
T5Ha	aaaaggcaatgcgtgaatct	
SNATs	aagccatagcttcagctcca	full-length cDNA cloning
SNATa	aattgcagcgctaaaccaat	
ASMTs	agctgctccagcaattgagt	full-length cDNA cloning
ASMTa	aagctgccagtgccatatta	
TDCs-real	aaccgggaagatttttgctt	real-time qRT-PCR
TDCa-real	cacatgcctctcctcagtca	
T5Hs-real	accctggcactaccaatctg	real-time qRT-PCR
T5Ha-real	caaatgccactcgacagaga	
SNATs-real	gatcagaggggaatgaccaa	real-time qRT-PCR
SNATa-real	gccctgataaccaggatcaa	
ASMTs-real	gatgagcctagctccaatgc	real-time qRT-PCR
ASMTa-real	ggcaaagtcccaaatctcaa	
*EF2*s	ggtgtgacgatgaagagtgatg	reference gene
*EF2*a	tgaaggagagggaaggtgaaag	
*actin*s	cttgcatccctcagcacctt	reference gene
*actin*a	tcctgtggacaatggatgga	
